# From Pellets to Snacks: Effects of Deep-Frying and Microwave Heating on Polyphenols, Physicochemical Properties and Sensory Profiles of Mushroom-Enriched Snacks

**DOI:** 10.3390/molecules31132256

**Published:** 2026-06-26

**Authors:** Agnieszka Nemś, Joanna Kolniak-Ostek, Anna Michalska-Ciechanowska, Artur Gryszkin, Agnieszka Kita

**Affiliations:** 1 Department of Food Storage and Technology, Faculty of Biotechnology and Food Sciences, Wroclaw University of Environmental and Life Sciences, 51-630 Wrocław, Poland; artur.gryszkin@upwr.edu.pl; 2 Department of Fruit, Vegetable and Plant Nutraceutical Technology, Faculty of Biotechnology and Food Science, Wrocław University of Environmental and Life Sciences, 51-630 Wrocław, Poland; joanna.kolniak-ostek@upwr.edu.pl (J.K.-O.); anna.michalska@upwr.edu.pl (A.M.-C.)

**Keywords:** mushrooms, pellet snacks, expanded products, bioactive compounds, food quality, functional ingredients

## Abstract

The aim of this study was to evaluate the effect of incorporating button mushroom (*Agaricus bisporus*) powder (5% and 10%, *w*/*w*) and two expansion methods (deep-fat frying and Fmicrowaving) on the nutritional, bioactive, sensory, and physical properties of third-generation snacks. Mushroom addition increased the contents of protein, raw fiber, ash and polyphenols compounds, particularly caffeic acid and chlorogenic acid derivatives. The highest nutritional value was observed in microwave-expanded snacks containing 10% mushroom powder, which showed increased protein (4.59%), ash (2.5%) and raw fiber (3.31%) contents combined with very low fat level (0.14%) Microwave expansion promoted better retention of bioactive compounds with the highest total polyphenol content reaching 195.48 mg/kg. Instrumental sensory analyses revealed that mushroom addition intensified bitter and metallic taste attributes and enhanced roasted and earthy aroma notes associated with increased levels of pyrazines, phenols, alcohols, and acids. Moreover, mushroom incorporation reduced expansion at higher inclusion levels, altered texture, and caused a darker color. Overall, dried mushroom powder proved to be an effective potential functional ingredient that improved the nutritional and antioxidant value of third-generation snacks, while microwave expansion offered superior retention of bioactive compounds and more favorable physical characteristics.

## 1. Introduction

Third-generation snacks (3G snacks), also known as pellet snacks, are semi-finished products typically obtained by extrusion cooking and characterized by a dense, non-expanded structure and low moisture content. Unlike directly expanded snacks, they require a subsequent expansion step, such as frying, baking, or microwave heating, to develop their final texture and sensory properties [1]. This two-stage production process provides flexibility in formulation and facilitates the incorporation of functional ingredients to enhance the nutritional value of the final product [2]. Mushrooms are increasingly recognized as valuable sources of polyphenolic compounds, including phenolic acids and flavonoid-like substances, which contribute significantly to their antioxidant capacity. These compounds are capable of scavenging reactive oxygen species, chelating pro-oxidant metal ions, and inhibiting oxidative processes, thereby reducing cellular oxidative stress. In addition to their antioxidant effects, mushroom polyphenols have been associated with anti-inflammatory, antimicrobial, antidiabetic, and anticancer activities [3]. These properties make them promising potential functional additives for snack formulations. Incorporation of mushroom powder into extruded snacks has been shown to increase protein, fiber, ash, polyphenol content, and free radical scavenging properties, while also influencing color intensity and texture properties [4].

Following extrusion, a secondary expansion process is required to produce the final crispy structure of pellet snacks [5]. Deep-fat frying remains a prevalent industrial method, generating rapid heat transfer, moisture evaporation, and characteristic flavor development, but often with substantial oil uptake and thermal exposure [6]. Microwave heating provides an alternative expansion technique based on volumetric dielectric heating of internal water molecules, enabling expansion without direct oil contact and often resulting in differing physicochemical outcomes compared to conventional frying. These disparities in heat-mass transfer can result in distinct effects on matrix porosity, expansion ratio, and retention of selected bioactives [7].

The choice of expansion technique critically influences the physicochemical properties of the final product, such as bulk density, expansion ratio, texture, and color, which are linked to starch gelatinization, matrix microstructure, and interactions with added functional ingredients as present in mushroom powder [8]. Moreover, the stability and extractability of phenolic compounds are affected by the processing conditions. Mushrooms are recognized as rich sources of phenolic acids and flavonoids, with diverse phenolic profiles documented across species [9].

In addition to physicochemical and compositional characteristics, sensory attributes such as aroma and taste are important determinants of consumer acceptance, particularly in mushroom-enriched products due to their distinctive flavor profile [10,11]. Consumer perception of innovative food products may vary across countries depending on familiarity with ingredients, and cultural preferences [12]. Therefore, both sensory evaluation and instrumental techniques, including electronic nose (e-nose) and electronic tongue (e-tongue) are increasingly used to characterize aroma and taste profiles and to differentiate products processed under various conditions. These tools have proven effective in assessing flavor characteristics of mushroom-based products and often show good agreement with human sensory evaluations [13].

Despite the growing interest in mushroom-based functional ingredients as well as advances in snack processing and sensory profiling technologies, there is a lack of comparative studies integrating basic chemical composition, polyphenol profiling, physicochemical characterization, and instrumental sensory analysis in the context of mushroom-enriched pellet snacks expanded by different methods. An evaluation of how expansion technique (deep-fat frying vs. microwave) affects both nutritional and sensory parameters is crucial for understanding the multi-faceted impact of processing technologies.

Therefore, the objective of this study was to compare the effects of deep-fat frying and microwave heating on mushroom-enriched pellet-based snacks, focusing on: (i) basic chemical composition, (ii) polyphenol content and profile, (iii) aroma and taste profiles assessed using electronic nose and electronic tongue technologies, and (iv) physicochemical properties including color, texture, degree of expansion and porosity.

## 2. Results and Discussion

### 2.1. Basic Chemical Composition of Mushroom-Enriched Snacks

The present study demonstrates that the incorporation of mushroom powder at both 5% and 10% levels significantly altered the proximate composition of the food matrix, with effects modulated by the expansion method applied (Table 1). Addition of mushrooms increased the total protein content in both fried and microwaved samples. The incorporation of 10% mushroom powder increased the protein content of the snacks regardless of the expansion method. In fried products, protein content increased from 3.13% in the control sample to 4.56%, while in microwave-expanded snacks it increased from 2.31% to 4.59%. That was consistent with the literature indicating that mushrooms contribute substantial protein and essential amino acids to food products, making them an effective nutrient-enriching ingredient in new formulations and dietary patterns [14]. Likewise, the observed increase in raw fiber with mushroom inclusion reflects the high dietary fiber content intrinsic to edible fungi, notably β-glucans and chitin fractions, which have been widely reported to improve digestive health and functional properties of enriched foods [15]. The addition of 10% mushroom powder increased crude fiber levels from 1.33% to 1.86% in fried snacks and from 1.43% to 3.31% in microwave-expanded products. The fat content was markedly higher in fried samples than in microwave-treated ones, underscoring the well-established effect of oil uptake during frying and contrasting sharply with the minimal fat accrual observed with microwave cooking, a trend similarly documented in studies comparing expanding methods on mushroom nutrient retention [16]. Mushroom-enriched snacks expanded by deep-frying contained on average 23.4% fat whereas those expanded by microwave treatment contained only 0.14% fat. Among the deep-fried samples, the incorporation of mushroom powder significantly increased fat content compared with the control sample (from 17.78%—control sample to 23.93%—5% of mushroom powder and 22.80%—10% mushroom powder). This effect may be attributed to the porous structure of mushroom tissues and the mass transfer phenomena occurring during frying, where water loss creates voids that can subsequently be filled by oil. Oil uptake in fried products is strongly influenced by food composition, moisture loss, pore development, and microstructural characteristics during frying and cooling phases [17]. Interestingly, the fried sample containing 10% mushroom powder exhibited a lower fat content than the sample with 5%. This phenomenon may be explained by structural modifications induced by the higher level of mushroom powder incorporation. Increased amounts of dietary fiber and other non-starch components may alter the expansion behavior and microstructure of the pellets, leading to the formation of a less interconnected pore network and reducing the availability of pathways for oil penetration [18,19]. Previous studies have demonstrated that oil absorption is closely related to pore size distribution, pore connectivity, and crust microstructure rather than simply to the total porosity of the product [20,21]. Furthermore, higher levels of fiber-rich ingredients may promote the formation of a more compact surface layer during frying, thereby limiting oil uptake, particularly during the cooling stage when a substantial proportion of oil is absorbed into the product structure [20]. Therefore, the lower fat content observed in samples containing 10% mushroom powder suggests that the effect of mushroom addition on oil absorption depends not only on its presence but also on the structural changes induced by its concentration. Moreover, variations in ash content suggest that mushrooms contribute additional minerals, aligning with nutritional models showing that even modest amounts of mushrooms can substantially increase mineral micronutrient intake without increasing sodium intake. The low total sugar levels across all mushroom-enriched treatments likely reflect the inherently low sugar content of mushroom biomass [22]. The slightly higher total sugar content observed in mushroom-enriched snacks after microwave expansion may be attributed to the milder processing conditions of microwave heating, which can reduce the consumption of sugars in Maillard and caramelization reactions compared with deep-fat frying. In addition, microwave treatment may enhance the disruption of the mushroom cellular matrix, increasing the extractability of soluble carbohydrate fractions. In contrast, the higher sugar content detected in the fried control sample may result from thermal degradation and partial depolymerization of starch during frying, leading to the formation of low-molecular-weight carbohydrate compounds measurable as sugars. Therefore, the observed differences are likely related to variations in carbohydrate transformation, sugar retention, and extractability during processing [23]. Importantly, the expansion method also influenced nutrient outcomes: while frying elevated fat and protein measures due to matrix changes during high-temperature lipid absorption, microwaving tended to preserve more native components and limited nutrient losses, corroborating research showing that microwave cooking better maintains protein and overall nutritional profiles compared with other thermal treatments such as boiling or deep-frying [16].

### 2.2. Polyphenol Profile and Its Relationship with Sensory Characteristics of Mushroom-Enriched Snacks

The addition of mushroom powder significantly affected both the profile and total content of polyphenolic compounds in the analyzed snacks, and the magnitude of these changes depended on both the level of mushroom incorporation and the expansion method used. The total content of identified polyphenols increased progressively with increasing mushroom addition, ranging from 26.9 to 33.65 mg/kg in control samples to 135.93–195.48 mg/kg in snacks containing 10% mushroom powder (Table 2). Compared with the fried control sample (26.9 mg/kg), microwave expansion of the control formulation resulted in a 25% higher total polyphenol content (33.65 mg/kg). The enrichment with 5% mushroom powder increased the total polyphenol concentration to 80.87 and 109.14 mg/kg in fried and microwave-expanded snacks, respectively, corresponding to approximately three- and four-fold increases relative to the fried control. The highest values were observed in snacks supplemented with 10% mushroom powder, reaching 135.93 mg/kg after frying and 195.48 mg/kg after microwave expansion. These values represent increases of approximately 5.1-fold and 7.3-fold, respectively, compared with the control product. Furthermore, at each supplementation level, microwave-expanded samples exhibited significantly higher total polyphenol contents than their fried counterparts, indicating that the processing method substantially influenced the measurable concentration of phenolic compounds in the final snacks.

Particularly high values were observed in microwave-expanded products. However, based on the present analytical data, it is not possible to distinguish whether the increased phenolic content resulted from improved retention of native phenolic compounds, enhanced extractability due to structural modifications of the food matrix, or a combination of both mechanisms. Therefore, the higher concentrations detected after microwave expansion should be interpreted as greater measurable phenolic content rather than unequivocal evidence of improved preservation. Similar observations were reported by Latif et al. [24], who demonstrated that microwave-assisted processes enhanced the extraction efficiency and release of phenolic compounds from the *Agaricus bisporus* matrix.

The polyphenolic profile was mainly dominated by caffeic acid derivatives and hydroxycinnamic acids, including caffeic acid, caffeic acid hexoside isomers, 1-caffeoylquinic acid, and dihydrocaffeic acid 3-O-glucuronide (Table 2). A particularly pronounced increase was observed for caffeic acid, whose concentration increased from approximately 8 mg/kg in the control samples to over 73 mg/kg in the microwave-expanded snacks containing 10% of mushroom powder. Similar trends were observed for chlorogenic acid derivatives and compounds such as (+)-catechin 3-O-gallate and quercetin derivatives, which were nearly absent in the control samples but appeared after mushroom incorporation. These findings are consistent with previous reports indicating that edible mushrooms are valuable sources of hydroxycinnamic acids, particularly caffeic, ferulic, and p-coumaric acid derivatives, which substantially contribute to the antioxidant potential of mushroom-based products [9].

The differences observed between the expansion methods can be explained by the distinct heat transfer mechanisms. During deep-fat frying, high temperatures and direct contact with oil may promote the partial thermal degradation and oxidation of the phenolic compounds. In contrast, microwave heating involves rapid volumetric energy transfer and shorter exposure to elevated temperatures, thereby limiting thermal degradation and potentially enhancing the release of compounds bound within the polysaccharide–protein matrix of the cell walls. Similar relationships were reported by Roncero-Ramos et al. [16], who demonstrated that microwave processing preserves bioactive compounds and the antioxidant capacity of cultivated mushrooms more effectively than conventional thermal treatments. From a technological and nutritional perspective, microwave expansion appears to be a promising alternative to deep-fat frying, as it results in a higher content of phenolic compounds while simultaneously limiting fat uptake. This may contribute to the development of mushroom-enriched snacks with improved functional value and lower caloric density.

**Figure 1:**
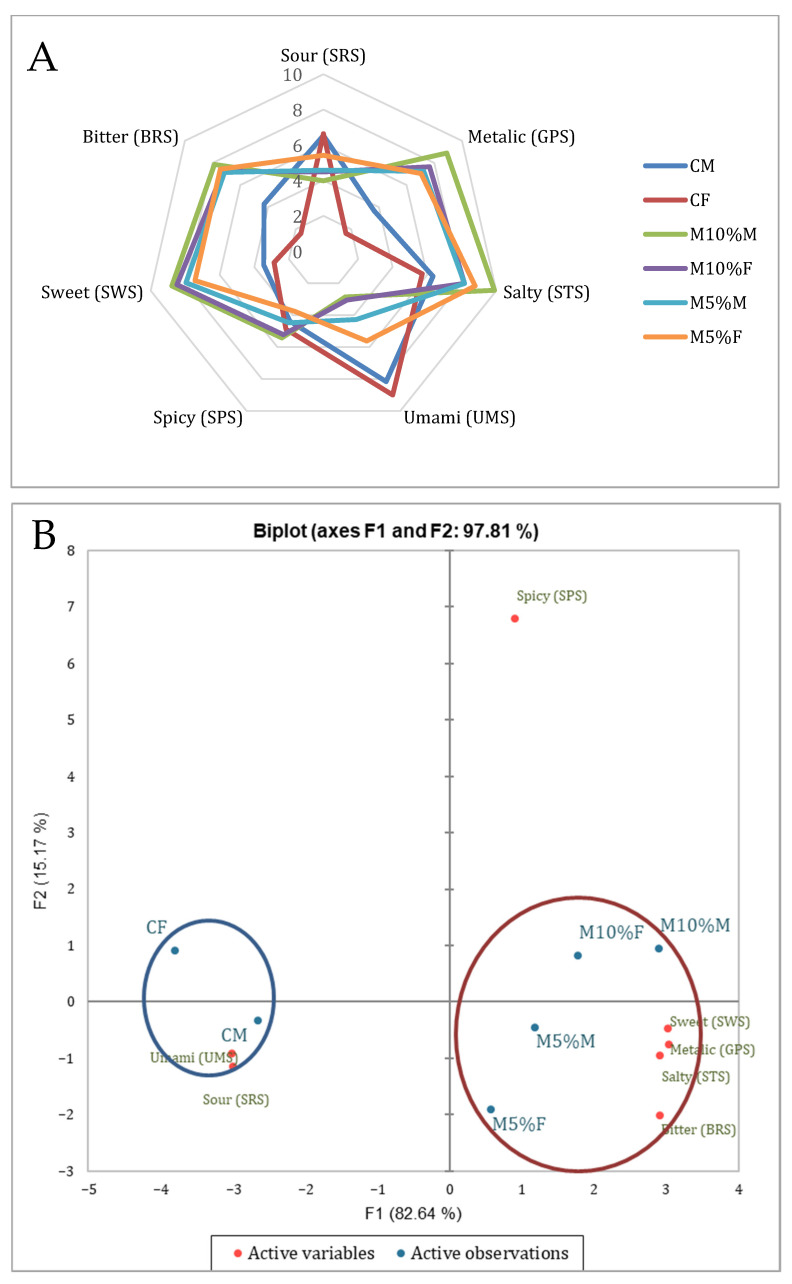
Radar plot showing the taste intensities of the analyzed snacks (**A**) and PCA graph showing the relationship among snacks samples and tastes (**B**). SRS—sour, GPS—metallic, STS—salty, SPS—spicy–piquant, UMS—umami, SWS—sweet, BRS—bitter for the following samples: CM—microwave control, CF—fried control, M10%M—10% mushroom microwave, M10%F—10% mushroom fried, M5%M—5% mushroom microwave, M5%F—5% mushroom fried.

Changes in the polyphenolic composition were also reflected in the instrumental sensory profiles by using an electronic tongue system (Appendix B). The addition of mushroom powder, which is rich in bioactive compounds and polyphenols resulted in higher bitterness (BRS) and metallic taste (GPS) intensities, accompanied by a reduction in umami perception (Figure 1A). This phenomenon may be associated with the presence of phenolic acids, particularly caffeic and chlorogenic acid derivatives, which are widely recognized as contributors to bitter and astringent taste sensations in different food products [25]. The highest bitterness and metallic scores were recorded for the samples containing 10% mushroom powder, which also exhibited the greatest total polyphenols levels.

The PCA graph showed that the first two principal components explain 97.81% of the total variability, with PC1 accounting for 82.64% and PC2 for 15.17%, respectively (Figure 1B). The samples showed a clear distribution between PC1: the control samples (fried-CF and microwaved-CM) are located on the negative side and are associated with sour and umami tastes, whereas the mushrooms samples (5% and 10%, both fried and microwaved) are positioned on the positive side and correlated with sweet, salty, metallic, bitter, and spicy sensations. Among the sensory descriptors, spiciness (SPS) is strongly associated with the positive direction of PC2, whereas bitterness (BRS) is linked to the negative direction of PC2. The close grouping of mushroom samples (5% and 10%) indicates that these treatments (frying and microwaving) produced similar sensory profiles, distinct from the control samples.

**Figure 2:**
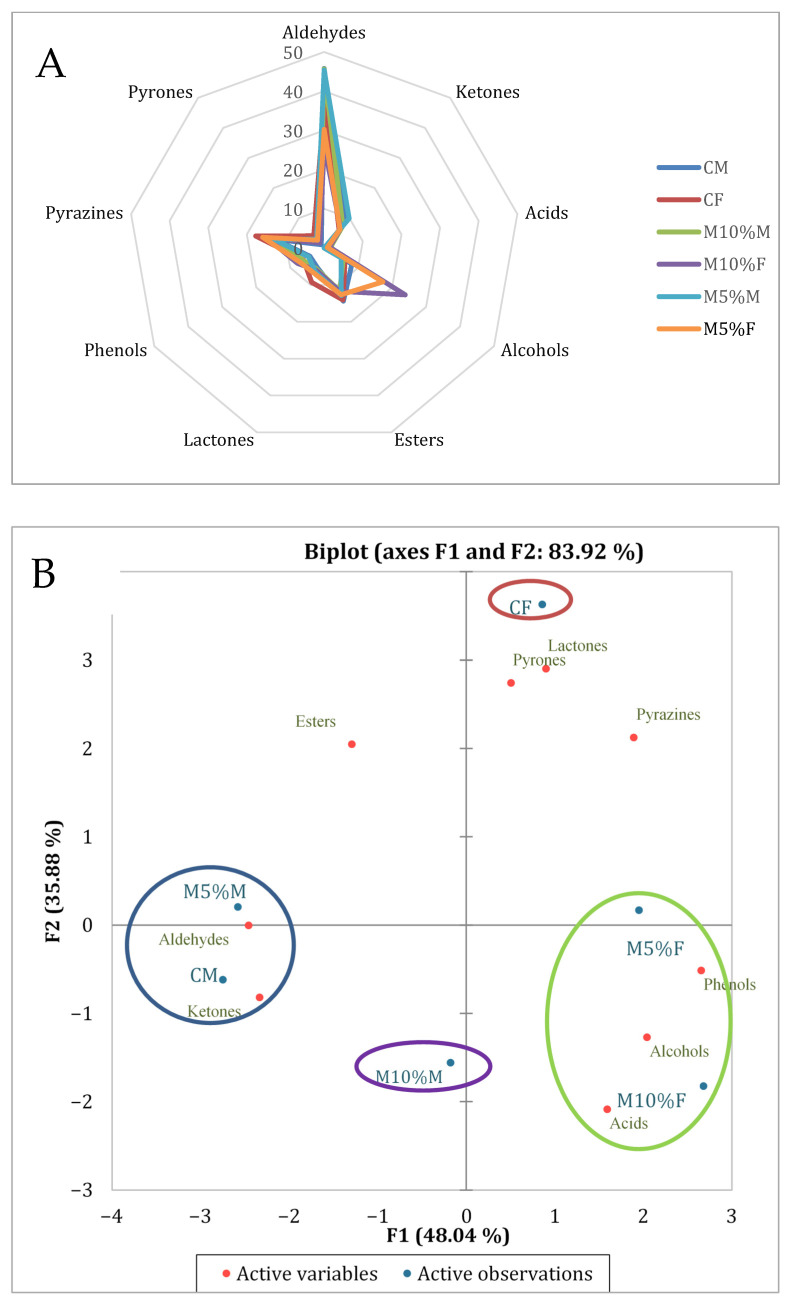
Radar plot showing the profiles of individual groups of aromatic compounds in the studied puffed snack samples (**A**) and PCA graph showing the relationship among snacks samples and groups of volatile compounds (**B**). (CM—control microwaved; CF—control fried; M10%M—mushrooms 10% microwaved; M10%F—mushrooms 10% fried; M5%M—mushrooms 5% microwaved; M5%F—mushrooms 5% fried).

An interesting relationship was also observed between the polyphenolic profile and the volatile compound composition evaluated using an electronic nose (Appendix A). The mushroom-enriched samples contained higher amounts of volatile compounds associated with Maillard reactions and thermal degradation processes, including pyrazines, furfural, and 4-ethylguaiacol (Figure 2A). These compounds are responsible for the roasted, earthy, caramel-like, and smoky aroma notes characteristic of thermally processed mushrooms and extruded products, respectively. Wei et al. [26] emphasized that pyrazines are among the most important volatile Maillard reaction products, contributing to roasted and nutty flavor attributes in heat-processed foods. Moreover, phenolic compounds may influence the formation of volatile aroma compounds during thermal processing through oxidative reactions and transformations of aroma precursors. The coexistence of elevated phenolic content and Maillard-derived volatiles suggests potential interactions between phenolic compounds and thermal reaction pathways during the expansion of the snack. Notably, the mushroom-enriched samples exhibited increased 4-ethylguaiacol content, a compound commonly associated with smoky and spicy notes in phenolic-rich thermally processed foods.

The PCA graph, showing the relationships among snack samples and groups of volatile compounds, indicated that the first two principal components explained 83.92% of the total variability, with PC1 accounting for 48.04% and PC2 for 35.88% (Figure 2B). The samples are separated mainly along PC1, distinguishing control snacks expanded by microwaving (CM) and mushroom snacks 5% expanded by the same method (M5%M) on the negative side from the fried control sample (CF) as well as fried snacks with mushrooms (M5%F and M10%F) on the positive side. Samples located on the positive side of PC1 were associated with higher levels of pyrazines, lactones, phenols, alcohols, and acids, whereas samples on the negative side were more closely related to aldehydes, ketones, and esters. Sample of fried control snacks (CF) is strongly associated with lactones and pyrones, while the fried 10% mushroom snacks (M10%F) are positioned near acids and alcohols, indicating a greater contribution of these compound groups. In contrast, microwaved control snacks (CM) and microwaved 5% mushroom snacks (M5%M) are clustered near aldehydes and ketones, suggesting a distinctly different volatile profile compared with the other samples.

### 2.3. Physical Quality Attributes of Mushroom-Enriched Snacks

Incorporation of mushroom powder into snack formulations markedly influenced instrumental color parameters, texture, and expansion characteristics (Table 3), with patterns consistent with prior reports on mushroom-enriched cereal and snack products. Moreover, the pellet expansion methods significantly affected the parameters of the obtained snacks. Frying induces expansion primarily through rapid surface heating and moisture evaporation at high temperatures (160–190 °C), leading to starch gelatinization, protein denaturation, and the formation of a rigid porous structure [27]. In contrast, microwave expansion is driven by volumetric dielectric heating, in which water molecules absorb microwave energy and generate internal steam pressure that expands the product from within, often resulting in a more homogeneous pore structure and lower lipid uptake [28].

Increasing the mushroom content in analyzed products led to a decrease in lightness (L*) and a shift toward more intense red–green (a*) and yellow–blue (b*) coordinates, resulting in darker and more saturated products than the control product without mushroom powder addition (Table 3). The color parameters clearly differentiated the two expansion methods. Fried samples exhibited significantly lower L* values and higher coordinate a* values than the microwave-expanded counterparts, particularly at higher mushroom inclusion levels. This indicates darker and redder products, which can be attributed to intensified Maillard reactions and increased pigment concentration during frying [12]. The presence of mushroom powder, which contains natural brown pigments and free amino acids, further enhances non-enzymatic browning reactions under high-temperature oil frying conditions. Similar reductions in lightness and increases in redness have been reported in mushroom-fortified extruded and fried snacks, where fiber-rich additives intensified thermal browning reactions and altered color perception [29,30]. In contrast, microwave-expanded snacks retained higher L* values, reflecting milder thermal conditions and the absence of surface oil-mediated heat transfer, consistent with previous findings on microwave-processed cereal-based snacks.

In terms of texture, mushroom addition at 5% and 10% levels reduced hardness in fried samples compared to control (Table 3), indicating a more brittle or crisp texture; this aligns with findings in mushroom-enriched products where dietary fiber and non-starch components disrupt the continuity of starch–protein matrices, leading to altered textural properties. Texture analysis further highlighted the contrasting structural effects of the two expansion techniques. Fried control samples showed higher hardness values, whereas mushroom addition significantly reduced hardness in fried snacks, especially at the 10% inclusion level. This reduction may result from disruption of the continuous starch matrix by mushroom dietary fiber, which weakens cell wall integrity and promotes a more brittle structure during rapid oil-induced expansion. Similar textural softening has been observed in fiber-enriched fried and extruded products, where non-starch polysaccharides interfere with starch–protein network formation [31]. In microwave-expanded samples, the texture values remained relatively stable across formulations, suggesting that volumetric heating produces a more uniform internal structure that is less sensitive to compositional changes. This supports previous reports indicating that microwave expansion yields products with lower mechanical resistance and more evenly distributed porosity [2].

The expansion index was significantly lower in the 10% fried mushroom sample, which may reflect impeded gas cell formation and matrix expansion due to the presence of non-starch constituents from mushrooms—a phenomenon previously reported in extruded snacks enriched with mushroom powder that exhibited reduced expansion and modified microstructure [32]. The expansion index was also strongly dependent on the expansion method. While the control and 5% mushroom powder-enriched snacks showed comparable expansion of processing method, a significant reduction in expansion was observed in the fried snacks containing 10% mushroom powder. This reduction is likely associated with the higher fiber content introduced by mushrooms addition, which limits bubble growth by restricting starch melt extensibility and steam cell expansion during frying. Additionally, oil penetration into the porous structure may stabilize the matrix prematurely, further reducing volumetric expansion. In contrast, microwave-expanded samples maintained relatively consistent expansion indices, even at higher mushroom levels, showing that internal steam generation is less hindered by fiber-rich ingredients. Similar trends have been reported in microwave-expanded starch-based systems, where expansion was less affected by fiber inclusion compared with conventional frying or extrusion processes [33].

**Figure 3:**
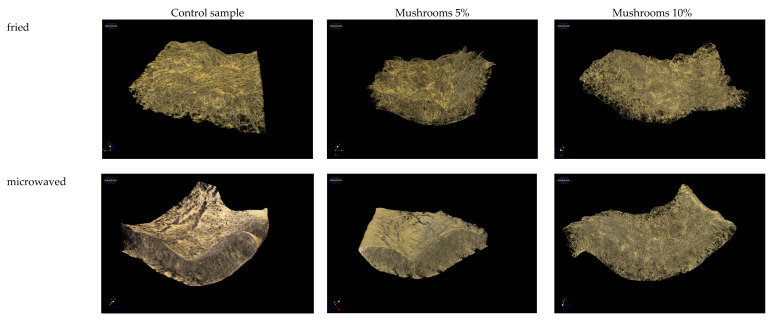
Three-dimensional micro-CT reconstructions of control and mushroom-enriched snack samples expanded by deep-fat frying and microwave heating.

The porosity of the snack samples ranged from 73.14% to 83.99% (Table 3). In both processing methods, the control samples exhibited the highest porosity values, reaching 83.99% for the fried snacks and 83.49% for the microwave-expanded snacks. The incorporation of mushroom powder resulted in a gradual decrease in porosity. In the fried samples, porosity decreased to 78.93% and 74.35% in samples containing 5% and 10% mushroom powder, respectively. A similar trend was observed in the microwave-expanded products, where porosity declined to 80.55% and 73.14% following the addition of 5% and 10% mushroom powder, respectively. The reduction in porosity with increasing mushroom powder content may be attributed to the presence of non-starch components, particularly dietary fiber and cell wall polysaccharides, which can interfere with starch gelatinization and expansion during processing. Fiber-rich ingredients are known to disrupt the continuity of the starch matrix, reduce bubble growth, and limit the expansion of extruded products, resulting in a denser internal structure and lower porosity values [34,35]. Similar reductions in expansion and porosity have been reported following the incorporation of fruit, vegetable, and other fiber-rich powders into starch-based extruded snacks [34]. The observed effect was more pronounced at the 10% addition level, indicating that increasing amounts of mushroom powder progressively restricted the development of the porous structure. Three-dimensional micro-CT reconstructions of control and mushroom-enriched snack samples expanded by deep-fat frying and microwave heating are presented in Figure 3.

Collectively, these results suggest that mushroom enrichment not only enhances nutritional value but also substantially alters physical quality attributes; the extent of these changes is modulated by both the level of mushroom addition and the selected processing method. Moreover, frying and microwave expansion generate distinct physicochemical pathways for structural development in mushroom-enriched snacks. Frying intensifies color development, increases structural variability, and is more susceptible to formulation changes, particularly fiber addition, while microwave expansion produces lighter-colored products with more stable textural and expansion characteristics and minimal fat uptake. From both technological and nutritional perspectives, microwave expansion appears to be more suitable method for producing mushroom-fortified snacks with consistent quality and improved nutritional value, whereas frying provides stronger sensory characteristics but results in higher fat content and greater sensitivity to formulation changes.

### 2.4. Principal Component Analysis (PCA)

**Figure 4:**
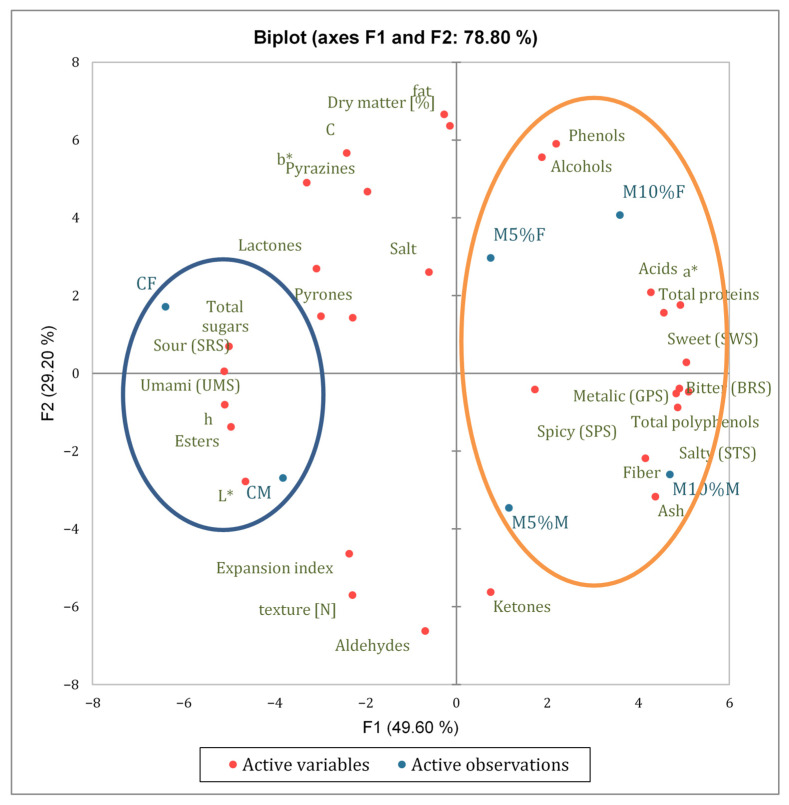
Principal component analysis (PCA) for snacks of control sample and samples with the addition of mushrooms (5% and 10%), expanded by frying in hot oil and by microwaving. (CM—control microwaved; CF—control fried; M10%M—mushrooms 10% microwaved; M10%F—mushrooms 10% fried; M5%M—mushrooms 5% microwaved; M5%F—mushrooms 5% fried).

Principal component analysis (PCA) of the combined e-nose, e-tongue, and physicochemical datasets further confirmed the clear differentiation of the samples according to the mushroom addition level and expansion method (Figure 4). Microwave-expanded samples containing 10% mushroom powder were strongly associated with high polyphenol content and intensified bitter and metallic taste profiles, whereas fried samples were characterized by a greater contribution of volatile compounds typical of intensive thermal reactions. These findings indicate that polyphenolic compounds may play an important role in determining the health-promoting potential of mushroom-enriched third-generation snacks and in shaping their sensory characteristics and overall flavor profile. Therefore, mushroom powder may serve as a natural source of antioxidant compounds and a multifunctional ingredient capable of modulating the sensory quality of third-generation snacks.

The results indicate a potential trade-off between the nutritional benefits and sensory quality of mushroom-enriched snacks. Although higher levels of mushroom powder increased the content of phenolic compounds and antioxidant potential, they were also associated with greater bitterness and metallic taste intensity. Since these sensory attributes may negatively affect consumer acceptance, optimization of mushroom concentration appears necessary to achieve a balance between enhanced functional value and desirable sensory properties. Future studies should therefore focus on identifying the maximum acceptable level of mushroom supplementation and exploring formulation strategies that could mitigate undesirable taste attributes while preserving the nutritional benefits.

## 3. Materials and Methods

### 3.1. Materials and Reagents

Mushrooms *Agaricus bisporus* were purchased in the local supermarket. Potato grits (ZPZ Lublin, Poland), potato starch (Melvit, Kruki, Poland), corn flour (Melvit, Kruki, Poland) were purchased online.

NaOH (Chempur, Piekary Sląskie, Poland, analytical grade), HCl (Chempur, Piekary Sląskie, Poland, analytical grade), Kiejdahl tablets (Sigma Aldrich, Steinheim, Germany), ether diethyl (Chempur, Piekary Sląskie, Poland, analytical grade), 3,5-dinitrosalicylic acid (Sigma-Aldrich, Steinheim, Germany, analytical grade). Formic acid and methanol were purchased from Sigma–Aldrich (Steinheim, Germany). Acetonitrile was purchased from Merck (Darmstadt, Germany). Quercetin 3-O-glucoside, quercetin 3-O-rutinoside, quinic, p-coumaric, and caffeoylquinic acid, and (+)-catechin were purchased from Extrasynthese (Lyon, France). Daidzin, caffeic and di-O-caffeoylquinic acid were purchased from Sigma–Aldrich (Steinheim, Germany).

### 3.2. Product Characteristics

**Figure 5:**
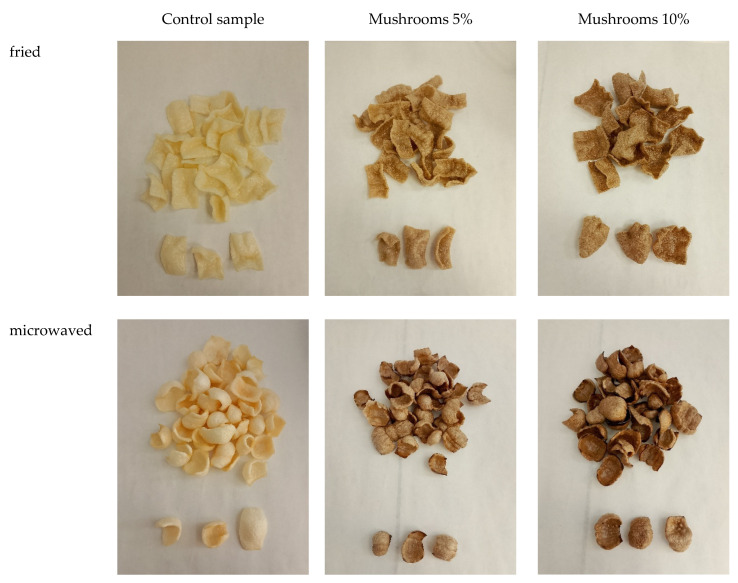
Representative photographs of control and mushroom-enriched snack samples prepared using different expanding methods.

The material used for the study was third-generation snacks (Figure 5) based on potato products (grits, starch), corn flour, and salt with the addition of dried champignons, *Agaricus bisporus* (mushroom powder), obtained according to the method described by Nemś et al. [12] in the amount of 5% and 10%. The control formulation consisted of 67% potato starch, 26% potato grits, 5% corn flour and 2% salt. The control sample was snacks without the addition of mushroom powder. Mushroom powder was added at concentration of 5% and 10% (*w*/*w*), based on total weight of the mixture. The formulation moisture was adjusted to 35–40% by adding the required amount of water. The hydrated mixture was subsequently homogenized, transferred into polyethylene bags, and kept under refrigerated conditions for 12 h to ensure uniform moisture distribution throughout the material. Pellets were obtained from the dough by low-temperature extrusion in a single-screw extruder (Brabender DN 20, Duisburg, Germany). The process parameters were set at a feed rate of 38 rpm, screw speed of 120 rpm, screw load of 2.8 A, die dimensions of 0.5 × 80 mm, and barrel temperatures of 60, 70, and 80 °C in consecutive heating zones [36,37]. The obtained non-expanded strands were cut into pellets of ca. 27 × 27 mm and dried at 20 ± 2 °C for 16 h on a laboratory table covered with tissue paper, under ambient laboratory conditions (relative humidity 40%) to approximately 10% moisture content and then sealed in polyethylene bags to equilibrate moisture until expansion. Pellet snacks were expanded from extruded pellets by frying in hot rapeseed oil at 185 °C for 15 s. The product to oil ratio was kept 1:20 (weight: volume; w:v) and by microwaving (800 W for 25 s) (Philips M 305, Seoul, Republic of Korea). Each microwave treatment batch consisted of 10 pellets, which were processed simultaneously under the selected operating conditions. All the expansion parameters were preselected experimentally. The obtained expanded snacks were stored at room temperature in closed plastic bags before analyses. The experiment was carried out in two technological replications.

### 3.3. Basic Chemical Composition

The contents of dry matter, total protein, fat, ash, crude fiber, and salt were determined following the official procedures of the Association of Official Analytical Chemists [38]. The dry matter was measured gravimetrically by drying the samples at 105 °C to constant mass. Protein content was calculated from total nitrogen determined by the Kjeldahl method using a Büchi Distillation Unit K-355 (Büchi Labortechnik AG, Flawil, Switzerland). A conversion factor of 6.25 was applied to convert nitrogen values into protein content [39]. The fat content was assessed using the Soxhlet extraction with diethyl ether as the extraction solvent in a Büchi B-811 extraction system (Flawil, Switzerland). The total ash content was determined by mineralization of the sample in a muffle furnace (PRODYN, Wodzisław Śląski, Poland) at 550 °C until complete combustion of organic matter was achieved. Total sugars content was quantified colorimetrically using the 3.5 dinitrosalicylic acid (DNS) method [40]. All analyses were performed in duplicate, and the results are presented as mean value (n = 2) ± standard deviation (SD).

### 3.4. Polyphenol Profile

Polyphenols were characterized by means of ultra-high-performance liquid chromatography combined with photodiode array detection and quadrupole time-of-flight mass spectrometry (UPLC-PDA-Q/ToF-MS) [41].

Samples were subjected to extraction with 80% ethanol (*v*/*v*), using a solvent-to-material ratio of 3:1. Following extraction, the suspension was filtered, sonicated for 20 min, centrifuged at 19,000× *g* for 10 min, and filtered through a 0.20 μm hydrophilic PTFE membrane (Millex Samplicity Filter, Darmstadt, Germany).

Chromatographic analyses were conducted on a UPLC system fitted with a BEH C18 analytical column (100 × 2.1 mm, particle size 1.7 μm) (Waters, Milford, MA, USA), thermostated at 30 °C. Separation was achieved using a binary solvent system composed of 0.1% formic acid in water (solvent A) and acetonitrile (solvent B). The elution gradient started with 99% solvent A and was maintained for 1 min, followed by a linear decrease to 65% solvent A over the next 11 min. Subsequently, the initial mobile-phase composition was restored within 0.5 min, and the column was re-equilibrated for an additional minute. The flow rate and injection volume were set at 0.42 mL min^−1^ and 5 μL, respectively.

Mass spectrometric measurements were carried out using an electrospray ionization interface operating in negative ionization mode. Spectra were acquired over the m/z range of 100–1500. Instrumental settings included a capillary voltage of 2.5 kV, cone voltage of 30 V, source temperature of 100 °C, and desolvation temperature of 300 °C. Nitrogen served as the desolvation gas throughout the analysis. Compound identification was established through the combined evaluation of exact mass data, chromatographic retention behavior, UV–Vis absorption characteristics, and tandem mass spectrometry fragmentation profiles, supported by comparison with authentic standards and published reports.

Quantitative determination was performed using external standard calibration curves exhibiting coefficients of determination (R^2^) above 0.99. In cases where pure reference compounds were unavailable, concentrations were estimated using calibration curves of structurally analogous compounds. Each sample was analyzed in triplicate to ensure analytical reliability.

### 3.5. Volatile Compounds Using an Electronic Nose (e-Nose)

The analyses were performed using an electronic nose system equipped with an HS-100 autosampler, a sensor array, and two columns operating in parallel: a non-polar column (MXT-5: 5% diphenyl, 95% methylpolysiloxane, 10 m length, 180 μm diameter) and a mid-polar column (MXT-1701: 14% cyanopropylphenyl, 86% methylpolysiloxane, 10 m length, 180 μm i.d.). Sample preparation involved weighing 1 g of homogenized material into 20 mL glass vials. After sealing with caps fitted with silicone–PTFE septa, the vials were placed on the autosampler tray of the HERACLES electronic nose (Alpha MOS, Toulouse, France) for analysis [42].

Volatile compounds were identified by comparing their retention times with those of a homologous series of n-alkane standards, allowing the calculation of Kovats retention indices (KI). The calculated retention indices were subsequently compared with reference values available in the AromaChemBase database (Alpha MOS, Toulouse, France) for compound identification.

Semi-quantitative analysis was performed using peak areas obtained from the flame ionization detector (FID) chromatograms, assuming a proportional relationship between detector response and analyte concentration. The results were expressed as the relative abundance (%) of individual volatile compounds. Each sample was analyzed in triplicate to ensure the repeatability of the measurements. Instrument control, data acquisition, and chromatographic data processing were carried out using AlphaSoft 14.2 software coupled with the AromaChemBase database (Alpha MOS, Toulouse, France).

### 3.6. Chemical Compounds Using an Electronic Tongue (e-Tongue)

The analyses were conducted using an electronic tongue system equipped with an Ag/AgCl electrode and seven potentiometric sensors (SRS, GPS, STS, UMS, SPS, SWS, and BRS), each exhibiting varying cross-sensitivities to specific compounds. The test samples, dissolved in appropriate solvents depending on the type of substance analyzed, were placed in the glass containers of the instrument’s autosampler and subjected to analysis. Water was used as a solvent. Using the AlphaSoft software (Alpha MOS, Toulouse, France) and the Taste Screening tool, the intensities of the following taste attributes were determined: sour (SRS), salty (STS), umami (UMS), sweet (SWS), bitter (BRS), metallic (GPS), and spicy–piquant (SPS) tastes. The intensity of each attribute was assessed on a scale of 0 to 12 arbitrary units (a.u.), with boundary values corresponding to undetectable and very intense [43]. All analyses were performed in triplicate (n = 3).

### 3.7. Color

The color characteristics of the snack samples were determined using a Minolta CM-5 spectrophotometer (Konica-Minolta, Osaka, Japan). Measurements were recorded in the CIE L* a* b* color system, where L* denotes lightness, a* represents the green (−) to-red (+) component. b* indicates the blue (−) to yellow (+) component. Based on the a* and b* values, chroma (C*) and hue angle (*h*°) were calculated. Chroma reflects the intensity or saturation of the color, while *hue angle* describes the dominant color tone and is expressed on a scale from 0° to 360°. Prior to analysis, the snacks were ground and measured against a white reference standard under laboratory conditions at 22 °C. Five measurements were performed for each sample, and the values for L*, a*, b*, *h*°, and *C* were averaged [44].

### 3.8. Texture

The texture of the obtained snacks was evaluated using an Instron 5544 universal testing machine (Instron corp. Norwood, MA, USA) controlled by BlueHill software (version 4.13). The maximum force (N) required to break the snack samples was determined with a shear blade at a test speed of 250 mm/min. Measurements were carried out at 22 °C and the maximum breaking force [N] was recorded. Results were reported as the mean values ± standard deviation (SD) based on 20 measurements [32].

### 3.9. Expansion Index

Snacks expansion was evaluated by measuring sample thickness before and after expansion. The thickness of each pellet was recorded in three locations using a digimatic caliper (Mitutoyo, Kawasaki, Japan) and the measurements were repeated after expansion. The expansion index was subsequently calculated from the ratio of the final to the initial thickness. Data were expressed as the mean value ± standard deviation (SD) based on 10 measurements.

### 3.10. Micro-Computed Tomography (Micro-CT) Analysis

Micro-computed tomography scanning was performed using a SkyScan 1272 micro-CT scanner (Bruker, Kontich, Belgium). A sample measuring 1.5 × 2 cm (width × height) was cut from the material using a razor blade and mounted vertically onto a metal stage. The scans were acquired at an X-ray source voltage of 50 kV and a current of 200 µA. The analysis was conducted with a rotation step of 0.3° and a spatial resolution of 13.0 µm. The total scanning time for a single sample was 51 min.

Reconstruction of the obtained images was performed across 1300 slices using NRecon software (version 1.6.10, Bruker, Kontich, Belgium). Sample cross-sections in the three orthogonal directions XYZ were obtained from the central part of the material using DataViewer software (version 1.5.2.6, Bruker, Kontich, Belgium). Three-dimensional (3D) visualization was carried out using CTVox software (version 3.3.0, Bruker, Kontich, Belgium). Image analysis and binarization within the threshold range of 24–1000 were performed using CTAn software, V. 1.23.0.2 (Bruker, Kontich, Belgium). The material porosity, pore size distribution, and wall thickness distribution were subsequently determined. The scans were performed in duplicate using two randomly selected samples.

### 3.11. Statistical Analysis

The effects of formulation (control, 5% mushroom powder and 10% mushroom powder) and expansion method (frying or microwave heating) on the basic chemical composition and polyphenol profile of the snacks were evaluated using a two-way analysis of variance (ANOVA), with formulation and expansion method as fixed factors. When significant effects were detected, Tukey’s honestly significant difference (HSD) test was applied for multiple comparisons between means.

Principal component analysis (PCA) was performed to explore relationships among samples and variables, as well as to visualize patterns of variation within the dataset.

Statistical significance was established at *p*-values < 0.05, unless otherwise specified. All statistical analyses were conducted using XLSTAT (XLSTAT Version, 2025.2.0, Addinsoft, Paris, France).

## 4. Conclusions

The present study demonstrated that the incorporation of mushroom powder (5% and 10%) into third-generation snack formulations significantly improved their nutritional and functional value by increasing the protein, dietary fiber, ash, and polyphenol (particularly hydroxycinnamic acid and caffeic acid derivatives) contents, thereby improving the antioxidant potential and supporting the use of edible mushrooms as functional ingredients in health-oriented foods.

The expansion method plays a crucial role in determining the final quality attributes of products. Compared with deep-fat frying, microwave expansion resulted in lower fat content, lighter color, more stable texture, and higher retention of polyphenol compounds, making it a more effective technology for preserving nutritional and functional properties.

The addition of mushrooms also markedly influenced the sensory-related parameters. Increased levels of mushroom powder were associated with higher bitterness and metallic taste intensities, likely due to the elevated concentration of phenolic compounds, while volatile analysis revealed enhanced formation of pyrazines and phenolic-derived aroma compounds characteristic of thermally processed mushroom products. The combined e-nose, e-tongue, and PCA analyses confirmed clear differentiation among samples according to mushroom concentration and processing method, demonstrating a strong relationship between chemical composition and sensory perception.

Overall, mushroom powder is a valuable multifunctional ingredient for the development of innovative and healthier third-generation snacks, with microwave expansion offering the most promising balance between nutritional quality, potential functional properties, and desirable physical characteristics.

Further research is recommended to improve the practical and industrial relevance of the developed snack products. In particular, consumer-based sensory studies involving target groups should be conducted to determine the most acceptable expansion method and the optimal level of mushroom powder addition. In addition, studies on shelf-life stability, microstructure, process scale-up, economic viability, and the potential for nutritional or functional claims would provide important insights supporting industrial implementation and commercialization of the product.

## Figures and Tables

**Table 1:** Chemical composition of snacks of the control sample and samples with the addition of dried mushrooms (5% and 10%), expanded by frying in hot oil (fried) and by microwaving (microwaved).

	Control Sample		Mushrooms 5%		Mushrooms 10%	
	Fried	Microwaved	Fried	Microwaved	Fried	Microwaved
Dry matter [g/100 g]	96.06 ± 0.10 ^bc^	95.09 ± 0.09 ^d^	96.50 ± 0.08 ^ab^	95.22 ± 0.15 ^cd^	97.07 ± 0.08 ^a^	94.51 ± 0.48 ^d^
Total proteins [g/100 g]	3.13 ± 0.07 ^c^	2.31 ± 0.03 ^d^	3.95 ± 0.08 ^b^	4.09 ± 0.13 ^b^	4.56 ± 0.09 ^a^	4.59 ± 0.06 ^a^
Fat [g/100 g]	17.78 ± 0.11 ^c^	0.17 ± 0.01 ^d^	23.93 ± 0.09 ^a^	0.14 ± 0.06 ^d^	22.80 ± 0.13 ^b^	0.14 ± 0.01 ^d^
Ash [g/100 g]	1.13 ± 0.03 ^c^	1.86 ± 0.07 ^b^	1.70 ± 0.07 ^bc^	2.01 ± 0.07 ^ab^	1.97 ± 0.06 ^ab^	2.50 ± 0.34 ^a^
Total sugars [g/100 g]	1.40 ± 0.07 ^a^	0.53 ± 0.03 ^b^	0.61 ± 0.06 ^b^	0.69 ± 0.04 ^b^	0.66 ± 0.11 ^b^	0.74 ± 0.04 ^b^
Raw fiber [g/100 g]	1.33 ± 0.06 ^d^	1.43 ± 0.10 ^cd^	2.02 ± 0.15 ^b^	2.08 ± 0.06 ^b^	1.86 ± 0.08 ^bc^	3.31 ± 0.16 ^a^
Salt [g/100 g]	1.40 ± 0.04 ^a^	1.50 ± 0.05 ^a^	1.55 ± 0.07 ^a^	1.21 ± 0.04 ^b^	1.40 ± 0.03 ^a^	1.44 ± 0.02 ^a^

Data are shown as mean± standard deviation of two replications (n = 2), and values followed by different superscript letters in the same row are significantly different (two-way ANOVA, Tukey’s HSD test, *p* < 0.05).

**Table 2:** Polyphenols profile determined by HPLC MS/MS of snacks from the control sample and samples with the addition of dried mushrooms (5% and 10%), expanded by frying in hot oil (fried) and by microwaving (microwaved).

Rt	MS	MS/MS	Compound	[mg/kg]					
				Control Sample		Mushrooms 5%		Mushrooms 10%	
				Fried	Microwaved	Fried	Microwaved	Fried	Microwaved
0.82	191.0077		Quinic acid	8.29 ± 0.25 ^c^	10.84 ± 0.30 ^b^	9.98 ± 0.85 ^b^	10.31 ± 0.74 ^b^	19.69 ± 0.88 ^a^	19.99 ± 0.57 ^a^
1.10	147.0331		Cinnamic acid	ND	ND	5.31 ± 0.04 ^d^	5.49 ± 0.05 ^c^	8.32 ± 0.04 ^b^	9.98 ± 0.06 ^a^
1.24	341.1470	179.0214	Caffeic acid hexoside isomer	1.33 ± 0.08 ^e^	3.68 ± 0.10 ^d^	10.24 ± 0.52 ^c^	13.48 ± 0.42 ^b^	13.51 ± 0.29 ^b^	19.53 ± 0.36 ^a^
1.45	357.1293	179.1258/135.0154	Dihydrocaffeic acid 3-O-glucuronide	ND	ND	14.12 ± 0.51 ^d^	14.72 ± 0.84 ^c^	23.28 ± 0.25 ^b^	28.44 ± 0.65 ^a^
1.59	341.0703	179.0573	Caffeic acid hexoside isomer	ND	ND	3.18 ± 0.02 ^d^	4.96 ± 0.03 ^c^	6.88 ± 0.02 ^b^	7.01 ± 0.02 ^a^
1.78	179.9080	135.0154	Caffeic acid	8.36 ± 0.11 ^e^	8.74 ± 0.10 ^e^	18.59 ± 0.21 ^d^	36.49 ± 0.10 ^b^	30.34 ± 0.15 ^c^	73.28 ± 0.56 ^a^
2.17	279.9854	163.0380	p-Coumaroyl malic acid	ND	ND	1.08 ± 0.01 ^d^	1.4 ± 0.01 ^c^	1.69 ± 0.01 ^b^	2.41 ± 0.02 ^a^
2.41	341.1236	191.0152	Caffeoylhexose unknown isomer	ND	ND	0.77 ± 0.01 ^d^	0.92 ± 0.01 ^c^	1.54 ± 0.02 ^b^	1.77 ± 0.01 ^a^
2.67	441.0261	219.0514/191.2156/173.1355	(+)-Catechin 3-O-gallate	ND	ND	1.73 ± 0.02 ^d^	3.46 ± 0.01 ^c^	6.13 ± 0.03 ^b^	6.23 ± 0.03 ^a^
3.00	325.1651	307.0325	p-Coumaric acid 4-O-glucoside	ND	ND	0.27 ± 0.01 ^d^	0.52 ± 0.01 ^c^	0.57 ± 0.01 ^b^	0.7 ± 0.01 ^a^
3.25	353.0812	191.1266/179.0334	1-Caffeoylquinic acid	5.49 ± 0.08 ^e^	5.68 ± 0.07 ^e^	9.95 ± 0.23 ^d^	11.17 ± 0.15 ^c^	14.56 ± 0.21 ^b^	15.32 ± 0.18 ^a^
3.82	337.0654	191.1256/163.0547	trans-5-p-Coumaroylquinic acid	ND	ND	0.15 ± 0.01 ^d^	0.25 ± 0.00 ^c^	0.45 ± 0.01 ^b^	0.5 ± 0.02 ^a^
4.03	353.0718	191.0410	5-Caffeoylquinic acid	2.5 ± 0.05 ^e^	2.59 ± 0.05 ^e^	2.75 ± 0.01 ^d^	2.88 ± 0.01 ^c^	4.62 ± 0.01 ^b^	5.12 ± 0.01 ^a^
4.71	367.2313	193.0697/173.1135	4-O-Feruloylquinic acid	0.1 ± 0.00 ^e^	0.35 ± 0.01 ^d^	0.5 ± 0.01 ^c^	0.5 ± 0.00 ^c^	0.73 ± 0.01 ^b^	1.23 ± 0.01 ^a^
6.60	463.1735	301.1268	Quercetin-3-O-glucoside	0.7 ± 0.01 f	1.49 ± 0.01 ^a^	0.74 ± 0.01 ^e^	0.81 ± 0.01 ^d^	1.24 ± 0.02 ^c^	1.3 ± 0.01 ^b^
8.52	771.1251	301.0352	Quercetin-O-hexosyl-O-rutinoside	0.05 ± 0.00 ^d^	0.1 ± 0.00 ^c^	0.05 ± 0.00 ^d^	0.11 ± 0.01 ^b^	0.12 ± 0.00 ^b^	0.19 ± 0.01 ^a^
8.65	529.1509	175.1533	Feruloylquinic acid hexoside	0.02 ± 0.00	0.03 ± 0.00	0 ± 0.00	0.01 ± 0.00	0.01 ± 0.00	0.01 ± 0.00
8.90	341.1046	179.0556	Caffeic acid hexoside isomer	0.07 ± 0.00 ^d^	0.14 ± 0.00 ^c^	0.28 ± 0.01 ^b^	0.3 ± 0.02 ^b^	0.29 ± 0.01 ^b^	0.41 ± 0.02 ^a^
9.31	449.1754	301.1048	Dihydroquercetin 3-O-rhamnoside	ND	ND	0.71 ± 0.01 ^d^	0.77 ± 0.02 ^c^	0.94 ± 0.01 ^b^	0.98 ± 0.01 ^a^
9.91	457.5059	253.1018	6″-O-Acetyldaidzin	ND	ND	0.46 ± 0.02 ^d^	0.55 ± 0.02 ^c^	0.97 ± 0.03 ^b^	1.03 ± 0.02 ^a^
10.74	227.2672	185.0215/143.0149	Trans-resveratrol	ND	ND	0.01 ± 0.00	0.04 ± 0.00	0.04 ± 0.00	0.05 ± 0.00
	Total			26.9 ± 1.15 ^f^	33.65 ± 1.35 ^e^	80.87 ± 2.36 ^d^	109.14 ± 3.15 ^c^	135.93 ± 2.27 ^b^	195.48 ± 2.98 ^a^

Data are shown as mean ± standard deviation of three replications (n = 3), and values followed by different superscript letters in the same row are significantly different (two-way ANOVA, Tukey’s HSD test, *p* < 0.05).

**Table 3:** Color, texture, expansion index and porosity of control snacks and snacks with the addition of dried mushrooms, expanded by frying in hot oil (fried) and by microwaving (microwaved).

	Control		Mushrooms 5%		Mushrooms 10%	
	Fried	Microwaved	Fried	Microwaved	Fried	Microwaved
L*	85.58 ± 0.26 ^b^	88.13 ± 0.05 ^a^	63.40 ± 0.48 ^e^	69.98 ± 0.21 ^c^	55.73 ± 0.37 ^f^	65.26 ± 0.35 ^d^
a*	0.96 ± 0.11 ^e^	1.70 ± 0.03 ^d^	4.17 ± 0.04 ^b^	3.65 ± 0.03 ^c^	5.28 ± 0.04 ^a^	4.29 ± 0.11 ^b^
b*	19.51 ± 0.24 ^a^	18.65 ± 0.10 ^b^	18.78 ± 0.09 ^b^	16.61 ± 0.10 ^c^	18.84 ± 0.15 ^b^	16.31 ± 0.30 ^c^
C*	19.54 ± 0.25 ^a^	18.72 ± 0.08 ^b^	19.24 ± 0.10 ^a^	17.00 ± 0.13 ^c^	19.57 ± 0.15 ^a^	16.87 ± 0.33 ^c^
*h*°	87.18 ± 0.33 ^a^	84.78 ± 0.08 ^b^	77.47 ± 0.08 ^c^	77.60 ± 0.10 ^c^	74.36 ± 0.10 ^e^	75.25 ± 0.10 ^d^
Texture [N]	33.34 ± 5.59 ^a^	34.91 ± 7.25 ^a^	22.34 ± 5.42 ^b^	33.54 ± 11.03 ^a^	21.13 ± 5.15 ^b^	33.13 ± 10.12 ^a^
Expansion index	2.94 ± 0.58 ^a^	3.01 ± 0.54 ^a^	2.92 ± 0.57 ^a^	2.98 ± 0.60 ^a^	2.47 ± 0.43 ^b^	2.93 ± 0.55 ^a^
Porosity [%]	83.99 ± 3.76 ^a^	83.49 ± 2.99 ^a^	78.93 ± 3.92 ^b^	80.55 ± 0.98 ^b^	74.35 ± 4.97 ^c^	73.14 ± 2.48 ^c^

Data are shown as mean± standard deviation of five replications (n = 5) for color parameters, twenty replication (n = 20) for texture, ten replication (n = 10) for expansion index, and two replication (n = 2) for porosity, and values followed by different superscript letters in the same row are significantly different (two-way ANOVA, Tukey’s HSD test, *p* < 0.05).

## Data Availability

Data supporting the findings of this study are available on request from the corresponding author.

## Appendix A

**Table A1:** Profile of aromatic compounds of control snacks and snacks with the addition of dried mushrooms, expanded by frying in hot oil (fried) and by microwaving (microwaved).

Compound	Control		Mushrooms 5%		Mushrooms 10%	
	Fried	Microwaved	Fried	Microwaved	Fried	Microwaved
Acetaldehyde	14.05 ± 0.18	17.53 ± 0.22	12.09 ± 0.16	21.92 ± 0.26	10.84 ±0.21	22.72 ± 0.15
propanal	8.62 ± 0.02	13.61 ± 0.04	5.03 ± 0.01	6.03 ± 0.02	2.75 ± 0.02	5.33 ± 0.02
2-propanol	6.14 ± 0.01	8.14 ± 0.02	4.17 ± 0.01	4.96 ± 0.02	2.91 ± 0.01	4.73 ± 0.02
2-methylpropanal	7.30 ± 0.02	6.68 ± 0.02	5.92 ± 0.01	8.22 ± 0.03	5.05 ± 0.04	6.73 ± 0.02
butane-2,3-dione	2.39 ± 0.01	3.87 ± 0.01	3.12 ± 0.01	4.53 ± 0.02	3.58 ± 0.01	4.25 ± 0.01
butan-2-one	1.84 ± 0.00	1.12 ± 0.00	0.72 ± 0.00	1.06 ± 0.00	0.00 ± 0.00	0.00 ± 0.00
acetic acid	0.00 ± 0.00	0.00 ± 0.00	0.85 ± 0.00	0.00 ± 0.00	1.39 ± 0.00	1.66 ± 0.01
3-methylbutanal	0.00 ± 0.00	1.11 ± 0.00	1.27 ± 0.00	3.61 ± 0.01	2.07 ± 0.00	4.99 ± 0.02
2,3-Pentanedione	0.95 ± 0.00	1.88 ± 0.00	1.78 ± 0.00	2.59 ± 0.01	2.31 ± 0.00	3.14 ± 0.01
Hexanal	0.00 ± 0.00	2.89 ± 0.01	0.00 ± 0.00	0.79 ± 0.00	0.00 ± 0.00	0.00 ± 0.00
furfural	6.68 ± 0.01	3.99 ± 0.01	5.59 ± 0.01	4.81 ± 0.02	4.90 ± 0.02	5.94 ± 0.02
2,6-Dimethylpyrazine	3.44 ± 0.01	2.60 ± 0.01	2.62 ± 0.00	1.90 ± 0.01	2.11 ± 0.01	2.87 ± 0.01
2,3-Dimethylpyrazine	0.00 ± 0.00	0.00 ± 0.00	0.42 ± 0.00	0.00 ± 0.00	0.69 ± 0.00	0.00 ± 0.00
2,5-Dimethylpyrazine	3.75 ± 0.01	2.54 ± 0.01	3.01 ± 0.01	2.68 ± 0.01	2.53 ± 0.01	3.30 ± 0.01
trimethylpyrazine	5.72 ± 0.01	3.61 ± 0.01	4.73 ± 0.01	3.19 ± 0.01	4.10 ± 0.01	4.32 ± 0.01
1-Octanol	0.00 ± 0.00	0.00 ± 0.00	5.54 ± 0.01	0.00 ± 0.00	9.08 ± 0.00	0.00 ± 0.00
benzyl alcohol	0.00 ± 0.00	0.00 ± 0.00	7.55 ± 0.01	0.00 ± 0.00	11.84 ± 0.00	0.87 ± 0.00
γ-lactone	2.92 ± 0.01	2.02 ± 0.01	2.51 ± 0.00	2.21 ± 0.01	2.26 ± 0.01	2.77 ± 0.01
2,3-Diethylpyrazine	0.00 ± 0.00	0.56 ± 0.00	0.00 ± 0.00	1.09 ± 0.00	0.00 ± 0.00	0.00 ± 0.00
Tetramethylpyrazine	3.43 ± 0.01	2.69 ± 0.01	4.27 ± 0.01	2.82 ± 0.01	4.81 ± 0.01	3.00 ± 0.01
Maltol	1.24 ± 0.00	0.59 ± 0.00	0.97 ± 0.00	0.67 ± 0.00	0.80 ± 0.00	0.79 ± 0.00
(E, Z)-2,6-nonadienal	0.00 ± 0.00	0.00 ± 0.00	0.39 ± 0.00	0.00 ± 0.00	0.64 ± 0.00	0.00 ± 0.00
ethyl maltol	2.86 ± 0.01	0.54±0.00	1.67 ± 0.00	2.30 ± 0.01	0.92 ± 0.00	1.09 ± 0.00
4-ethylguaiacol	6.08 ± 0.01	4.20 ± 0.01	6.34 ± 0.01	4.53 ± 0.02	6.51 ± 0.01	5.39 ± 0.02
gamma-nonalactone	0.83 ± 0.00	0.00 ± 0.00	0.32 ± 0.00	0.69 ± 0.00	0.00 ± 0.00	0.00 ± 0.00
2-Isobutyl-3-methoxy pyrazine	1.40 ± 0.00	0.85 ± 0.00	0.87 ± 0.00	1.25 ± 0.00	0.53 ± 0.00	0.81 ± 0.00
2-phenylethyl butanoate	3.90 ± 0.01	3.77 ± 0.01	4.22 ± 0.01	1.70 ± 0.01	4.43 ± 0.01	2.92 ± 0.01
Pentyl octanoate	5.12 ± 0.01	3.63 ± 0.01	4.56 ± 0.01	4.02 ± 0.01	4.21 ± 0.01	3.82 ± 0.01
delta-decalactone	2.96 ± 0.01	2.08 ± 0.01	2.13 ± 0.00	2.42 ± 0.01	1.61 ± 0.01	1.77 ± 0.01
delta-Nonalacton	1.24 ± 0.00	1.12 ± 0.00	1.47 ± 0.00	1.19 ± 0.00	1.61 ± 0.00	1.12 ± 0.00
Ethyl dodecanoate	0.00 ± 0.00	1.48 ± 0.00	0.00 ± 0.00	1.38 ± 0.00	0.00 ± 0.00	0.00 ± 0.00
Guaiol	0.00 ± 0.00	0.00 ± 0.00	0.80 ± 0.00	0.00 ± 0.00	1.32 ± 0.00	0.79 ± 0.00
Dodecan-4-olide	1.34 ± 0.00	0.00 ± 0.00	0.52 ± 0.00	0.00 ± 0.00	0.50 ± 0.00	0.00 ± 0.00
Benzophenone	0.76 ± 0.00	1.41 ± 0.00	0.66 ± 0.00	1.69 ± 0.01	0.60 ± 0.00	0.00 ± 0.00
Benzyl benzoate	3.03 ± 0.01	2.79 ± 0.01	2.47 ± 0.00	2.86 ± 0.01	2.11 ± 0.01	2.69 ± 0.01
Ethyl tetradecanoate	1.13 ± 0.00	1.78 ± 0.00	0.78 ± 0.00	1.73 ± 0.01	0.55 ± 0.00	1.36 ± 0.00
Benzyl phenyl acetate	0.88 ± 0.00	0.90 ± 0.00	0.62 ± 0.00	1.15 ± 0.00	0.45 ± 0.00	0.83 ± 0.00
	100.00 ± 0.00	100.00 ± 0.00	100.00 ± 0.00	100.00 ± 0.00	100.00 ± 0.00	100.00 ± 0.00

## Appendix B

**Table A2:** Results of the intensity assessment of sour, metallic, salty, umami, spicy, sweet, and bitter tastes in snack samples using the electronic tongue system and the Taste Screening tool.

Sample	Control		Mushrooms 5%		Mushrooms 10%	
	Fried	Microwaved	Fried	Microwaved	Fried	Microwaved
Sour (SRS)	6.64 ± 0.11	6.54 ± 0.11	5.42 ± 0.08	4.58 ± 0.08	4.48 ± 0.08	3.98 ± 0.08
Metalic (GPS)	1.62 ± 0.04	3.66 ± 0.05	7.04 ± 0.11	7.24 ± 0.11	7.66 ± 0.11	8.9 ± 0.16
Salty (STS)	5.72 ± 0.08	6.34 ± 0.11	8.78 ± 0.13	8.16 ± 0.11	8.16 ± 0.11	9.90 ± 0.16
Umami (UMS)	9.00 ± 0.16	8.16 ± 0.11	5.62 ± 0.08	4.28 ± 0.08	3.06 ± 0.05	2.86 ± 0.05
Spicy (SPS)	4.88 ± 0.08	4.38 ± 0.08	3.76 ± 0.05	4.48 ± 0.08	5.22 ± 0.08	5.42 ± 0.08
Sweet (SWS)	2.86 ± 0.05	3.46 ± 0.05	7.44 ± 0.11	7.96 ± 0.11	8.48 ± 0.13	8.78 ± 0.13
Bitter (BRS)	1.62 ± 0.04	4.28 ± 0.08	7.44 ± 0.11	7.14 ± 0.11	7.24 ± 0.11	7.86 ± 0.11
